# Smoking in Relation to Coronary Atherosclerotic Plaque Burden, Volume and Composition on Intravascular Ultrasound

**DOI:** 10.1371/journal.pone.0141093

**Published:** 2015-10-22

**Authors:** Nermina Buljubasic, K. Martijn Akkerhuis, Sanneke P. M. de Boer, Jin M. Cheng, Hector M. Garcia-Garcia, Mattie J. Lenzen, Rohit M. Oemrawsingh, Linda C. Battes, Melissa Rijndertse, Evelyn Regar, Patrick W. Serruys, Robert-Jan van Geuns, Eric Boersma, Isabella Kardys

**Affiliations:** Department of Cardiology, Clinical Epidemiology Unit, Erasmus MC, Rotterdam, the Netherlands; University of Bologna, ITALY

## Abstract

**Background:**

This study aimed to evaluate the relationship between cigarette smoking and coronary atherosclerotic burden, volume and composition as determined in-vivo by grayscale and virtual histology (VH) intravascular ultrasound (IVUS).

**Methods and Results:**

Between 2008 and 2011, (VH-)IVUS of a non-culprit coronary artery was performed in 581 patients undergoing coronary angiography. To account for differences in baseline characteristics, current smokers were matched to never smokers by age, gender and indication for catheterization, resulting in 280 patients available for further analysis. Coronary atherosclerotic plaque volume, burden, composition (fibrous, fibro-fatty, dense calcium and necrotic core) and high-risk lesions (VH-IVUS derived thin-cap fibroatheroma (TCFA), plaque burden ≥70%, minimal luminal area ≤4.0 mm^2^) were assessed. Cigarette smoking showed a tendency towards higher coronary plaque burden (mean±SD, 38.6±12.5% in current versus 36.4±11.0% in never smokers, p = 0.080; and odds ratio (OR) of current smoking for plaque burden above versus below the median 1.69 (1.04–2.75), p = 0.033). This effect was driven by an association in patients presenting with an acute coronary syndrome (ACS) (current smokers, plaque burden 38.3±12.8% versus never smokers, plaque burden 35.0±11.2%, p = 0.049; OR 1.88 (1.02–3.44), p = 0.042). Fibrous tissue tended to be lower in current smokers (mean±SD, 57.7±10.5% versus 60.4±12.6%, p = 0.050) and fibro-fatty tissue was higher in current smokers (median[IQR], 9.6[6.0–13.7]% versus 8.6[5.8–12.2]%, p = 0.039). However, differences in percentage necrotic core and dense calcium could not be demonstrated. Also, no differences were found with regard to high-risk lesions.

**Conclusions:**

An association between smoking and degree of coronary atherosclerosis was present in patients undergoing coronary angiography who presented with ACS. Although smoking was associated with higher fibro-fatty percentage, no associations could be demonstrated with percentage necrotic core, nor with VH-IVUS derived TCFA lesions. Since the magnitude of the differences in both degree and composition of atherosclerosis was modest, clinical relevance of the findings may be questioned.

## Introduction

Cigarette smoking is a well-known risk factor for developing coronary artery disease (CAD). Previous epidemiologic studies have demonstrated that cigarette smoking is associated with severity of atherosclerosis on both coronary angiography and coronary CT angiography [1,2], increased risk of myocardial infarction [3] and cardiovascular death [4,5].

In line with the above, several pathophysiologic effects of cigarette smoke exposure on cardiovascular function have been described. Both active and passive cigarette smoke exposure have been shown to promote endothelial dysfunction, stimulate inflammatory processes at the vessel wall and enhance vascular prothrombotic effects [6,7]. Thus, ample fundamental research evidence is available demonstrating that smoking directly impacts multiple aspects of atherosclerosis. However, less is currently known about the associations of smoking with in-vivo, macroscopic plaque composition and plaque vulnerability. Although coronary angiography enables evaluation of the unobstructed part of the lumen, it does not provide information on the structure of the arterial wall itself. Grayscale intravascular ultrasound (IVUS) also provides limited information on plaque characteristics.

Virtual histology (VH)-IVUS of the coronary arteries allows spectral analysis of backscattered radiofrequency ultrasound signal and herewith enables in-vivo analysis of the composition of atherosclerotic plaque as well as identification of thin-cap fibroatheroma (TCFA) lesions [8]. Until now, the association between smoking and in-vivo coronary plaque composition has only been examined in two studies. The first [9,10] applied VH-IVUS and examined several plaque components, but did not assess VH-IVUS derived TCFA. The second [11] used integrated backscatter IVUS, which is based on the same principle as VH-IVUS, but examined 30 patients only.

The main objective of the current study is to evaluate the relationship between cigarette smoking and coronary atherosclerotic plaque burden, volume and composition as assessed by (VH-)IVUS, including VH-IVUS derived TCFA lesions, in patients undergoing coronary catheterization for stable coronary artery disease (CAD) or acute coronary syndrome (ACS). With this investigation we aim to improve our understanding of the complex pathophysiologic relation between cigarette smoke exposure and cardiovascular disease.

## Methods

### Study population and baseline characteristics

This study was performed within the framework of the European collaborative Project on Inflammation and Vascular Wall Remodeling in Atherosclerosis—Intravascular Ultrasound (ATHEROREMO-IVUS) study. The design of the ATHEROREMO-IVUS study has been described in detail elsewhere [12]. In brief, 581 patients who underwent diagnostic coronary angiography or percutaneous coronary intervention (PCI) for ACS or stable CAD have been included in this study between 2008 and 2011 at Erasmus MC, Rotterdam, the Netherlands. The ATHEROREMO-IVUS study has been approved by the human research ethics committee of the Erasmus MC. Written informed consent was obtained from all participants. The study is registered in ClinicalTrials.gov, number NCT01789411.

Baseline characteristics of the patients, including smoking status, were prospectively entered into a dedicated database. Smoking status was determined by self-report. Patients were categorized into those who currently smoke cigarettes (including those that had quit less than 1 year ago), those who had never smoked, and those who had smoked in the past (and had quit more than 1 year ago). For the current sub-study, patients from the full ATHEROREMO-IVUS study cohort were eligible when they were current or never smokers. Patients who had quit smoking more than 1 year ago (n = 104), or for whom information on smoking was lacking (n = 1), were excluded, leaving 476 patients eligible for analysis.

### Intravascular ultrasound

Following the standard coronary angiography or PCI procedure, IVUS imaging of a non-culprit coronary artery was performed. The predefined order of preference for selection of the non-culprit vessel was: 1. left anterior descending (LAD) artery; 2. right coronary artery (RCA); 3. left circumflex (LCX) artery. All IVUS data were acquired with the Volcano^™^ s5/s5i Imaging System (Volcano Corp., San Diego, CA, USA), using a Volcano^™^ Eagle Eye^™^ Gold IVUS catheter (20 MHz). An automatic pullback system was used with a standard pull back speed of 0.5 mm per second. IVUS images were analyzed offline by an independent core laboratory (Cardialysis BV, Rotterdam, the Netherlands) that had no knowledge of clinical data. IVUS grayscale and virtual histology analyses were performed using pcVH 2.1 and qVH (Volcano Corp., San Diego, CA, USA) software.

The external elastic membrane and luminal borders were contoured for each frame (median interslice distance, 0.40 mm). Degree and phenotype of the atherosclerotic plaque were assessed. Plaque volume was defined as the percent of the volume of the external elastic membrane occupied by atheroma, i.e. percent atheroma volume. Plaque burden was defined as plaque and media cross-sectional area divided by external elastic membrane cross-sectional area. A coronary lesion was defined as a segment with a plaque burden of more than 40% in at least 3 consecutive frames. Using VH-IVUS, the composition of the atherosclerotic plaques was characterized into 4 different tissue types: fibrous (FI), fibro-fatty (FF), dense calcium (DC) and necrotic core (NC) [13]. These tissue type components were expressed as percentages of total plaque volume. Three types of high-risk lesions were identified: 1. VH-IVUS derived thin-cap fibroatheroma (TCFA) lesion, defined as a lesion with presence of >10% confluent necrotic core in direct contact with the lumen; 2. Lesion with large plaque burden, defined as a lesion with a plaque burden of ≥70%; 3. Stenotic lesion, defined as a lesion with a minimal luminal area of ≤4.0 mm^2^ [13,14]. In addition, remodeling index was calculated and expressed as the external elastic membrane cross-sectional area at the site of minimal luminal area divided by the reference external elastic membrane cross-sectional area. The reference site was selected <10 mm proximal to the lesion. Positive remodeling (arterial expansion) was defined as a remodeling index of >1.05, and negative remodeling (arterial shrinkage) was defined as a remodeling index of <0.95.

### Statistical analysis

Categorical data are presented as numbers and percentages. Normality of the distributions of continuous variables was examined by visual inspection of the histogram and by normal Q-Q plots. Continuous data are presented as mean±standard deviation (SD) or as median and interquartile range (IQR), depending on their distribution. Plaque volume, percentage fibro-fatty volume (% FF) and percentage dense calcium volume (% DC) appeared to be non-normally distributed and were therefore ln-transformed for further analyses.

Baseline clinical and procedural characteristics of current smokers and those who had never smoked were compared using the independent Student’s t-test for continuous variables and using the χ² test for categorical variables. Subsequently, to account for differences in baseline characteristics between current smokers and those who had never smoked, we performed a matching procedure. Every current smoker was matched to a never smoker by age (±5 years), gender and indication for catheterization (acute coronary syndrome or stable angina pectoris).

In the matched set, baseline clinical, procedural and (VH-)IVUS characteristics of current smokers and never smokers were compared using the paired samples t-test for continuous variables and the McNemar test or marginal homogeneity test for categorical variables, whichever was appropriate.

Subsequently, we performed conditional logistic regression to examine the associations between smoking status and high plaque burden (above versus below the median), as well as smoking status and the three types of high-risk lesions (VH-IVUS derived TCFA, lesion with plaque burden ≥70%, lesion with minimal luminal area ≤4.0 mm^2^).

Finally, to examine effect modification by age and indication for catheterization, we stratified on these variables and repeated all the above described analyses in subgroups. For this purpose, we divided age into tertiles (based on age of the smokers).

All data were analyzed with SPSS software (SPSS 20.0, IBM corp., Armonk, NY, USA). All statistical tests were two-tailed and p-values <0.050 were considered statistically significant.

## Results

### Baseline characteristics

Baseline clinical and procedural characteristics of the total patient population are presented in Table 1. Current (n = 169) and never smokers (n = 307) differed significantly at baseline. Current smokers, on average, were significantly younger (55.7±10.8 years vs. 64.4±10.8, p<0.001) than the never smokers. Significantly more men were present among the current smokers (79.3% vs. 70.7%, p = 0.041), and current smokers were less likely to have predisposing risk factors such as hypertension (p<0.001), dyslipidemia (p = 0.030) and diabetes mellitus (p = 0.038). Furthermore, the indication for coronary angiography or PCI also differed significantly between the two groups. Current smokers more often underwent catheterization for ACS and less often for stable CAD compared to the never smokers (p<0.001).

**Table 1:** Baseline clinical and procedural characteristics, before matching.

	Current smokers	Never smokers	P-value
	(n = 169)	(n = 307)	
**Patient characteristics**			
Age, years	55.7 ± 10.8	64.4 ± 10.8	<0.001
Male gender, n (%)	134 (79.3)	217 (70.7)	0.041
Hypertension, n (%)	63 (37.5)	171 (55.7)	<0.001
Dyslipidemia, n (%)	80 (47.6)	178 (58.0)	0.030
Diabetes mellitus, n (%)	20 (11.8)	59 (19.2)	0.038
Positive family history, n (%)	86 (51.2)	158 (51.5)	0.95
Peripheral artery disease, n (%)	12 (7.1)	15 (4.9)	0.32
Previous MI, n (%)	38 (22.5)	103 (33.6)	0.011
Previous PCI, n (%)	37 (21.9)	103 (33.6)	0.008
Previous CABG, n (%)	1 (0.6)	12 (3.9)	0.034
Previous stroke, n (%)	4 (2.4)	16 (5.2)	0.14
History of renal insufficiency, n (%)	8 (4.7)	17 (5.5)	0.71
**Procedural characteristics**			
*Indication for catheterization*			<0.001
Acute coronary syndrome, n (%)	119 (72.1)	151 (49.5)	
Stable angina pectoris, n (%)	46 (27.9)	154 (50.5)	
*Coronary artery disease*			0.24
No significant stenosis, n (%)	9 (5.3)	27 (8.8)	
1-vessel disease, n (%)	90 (53.3)	151 (49.2)	
2-vessel disease, n (%)	56 (33.1)	91 (29.6)	
3-vessel disease, n (%)	14 (8.3)	38 (12.4)	
*Vessel imaged by VH-IVUS*			0.15
LAD	71 (42.0)	101 (33.1)	
RCA	44 (26.0)	95 (31.1)	
LCX	54 (32.0)	109 (35.7)	

Values are mean ± SD or n (%). P-values were obtained by independent samples t-test or Chi-squared test, whichever was appropriate.

After the matching procedure, baseline clinical and procedural characteristics were similarly distributed between the two groups (Table 2).

**Table 2:** Baseline clinical and procedural characteristics, after matching.

	Current smokers	Never smokers	P-value
	(n = 140)	(n = 140)	
**Patient characteristics**			
Age, years	57.9 ± 9.7	58.1 ± 9.5	MV
Male gender, n (%)	108 (77.1)	108 (77.1)	MV
Hypertension, n (%)	58 (41.4)	73 (52.1)	0.10
Dyslipidemia, n (%)	74 (52.9)	74 (52.9)	1.00
Diabetes mellitus, n (%)	19 (13.6)	28 (20.0)	0.21
Positive family history, n (%)	68 (48.9)	75 (53.6)	0.53
Peripheral artery disease, n (%)	12 (8.6)	7 (5.0)	0.36
Previous MI, n (%)	33 (23.6)	35 (25.0)	0.89
Previous PCI, n (%)	33 (23.6)	45 (32.1)	0.11
Previous CABG, n (%)	0 (0.0)	3 (2.1)	0.25
Previous stroke, n (%)	4 (2.9)	7 (5.0)	0.51
History of renal insufficiency, n (%)	6 (4.3)	11 (7.9)	0.33
**Procedural characteristics**			
*Indication for catheterization*			MV
Acute coronary syndrome, n (%)	96 (68.6)	96 (68.6)	
Stable angina pectoris, n (%)	44 (31.4)	44 (31.4)	
*Coronary artery disease*			0.33
No significant stenosis, n (%)	6 (4.3)	14 (10.0)	
1-vessel disease, n (%)	74 (52.9)	76 (54.3)	
2-vessel disease, n (%)	47 (33.6)	36 (25.7)	
3-vessel disease, n (%)	13 (9.3)	14 (10.0)	
*Vessel imaged by VH-IVUS*			0.51
LAD	56 (40.0)	50 (35.7)	
RCA	40 (28.6)	43 (30.7)	
LCX	44 (31.4)	47 (33.6)	

MV, matching variable. Values are mean ± SD or n (%).

P-values were obtained by paired samples t-test, McNemar test or Marginal Homogeneity, whichever was appropriate.

### Degree of coronary atherosclerosis

**Fig 1:**
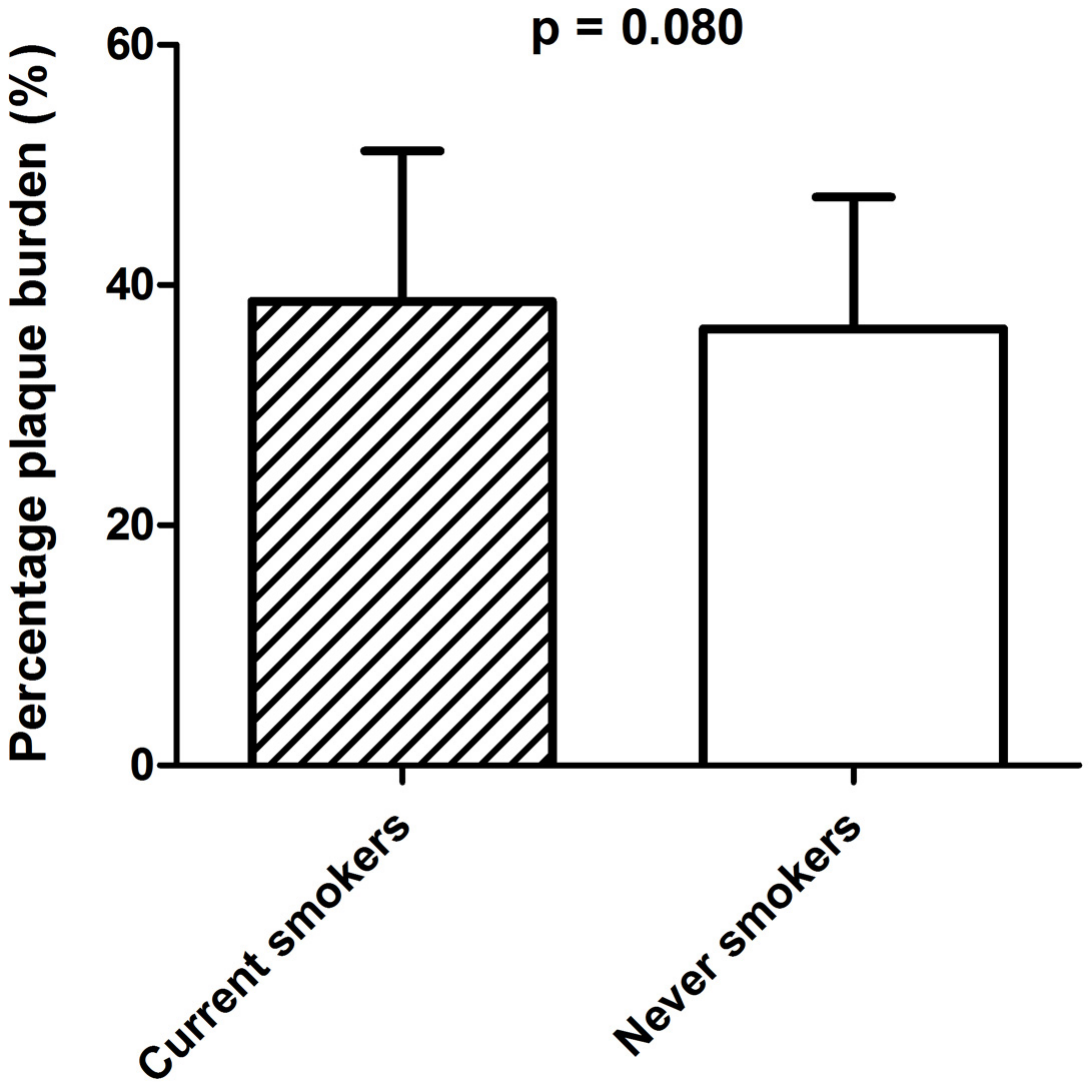
Difference in plaque burden between current and never smokers.

To assess differences in degree of atherosclerosis between current smokers and never smokers, plaque volume and plaque burden were examined in the coronary segments. Plaque volume (median (IQR)) was similar for current and never smokers (221.8[134.6–312.5]mm^3^ versus 207.5[134.5–293.2]mm^3^) (Table 3). On the other hand, with regard to plaque burden, there was a tendency towards higher values in current smokers (Table 3). Plaque burden (mean±SD) was 38.6±12.5% in current smokers versus 36.4±11.0% in never smokers, p = 0.080 (Fig 1).

**Table 3:** (VH-)IVUS segment and lesion characteristics, after matching.

	Current smokers	Never smokers	P-value
	(n = 140)	(n = 140)	
**(VH-)IVUS segment parameters**			
Segment length, mm	45.4 ± 15.4	44.7 ± 13.2	0.67
*Degree of atherosclerosis*			
Plaque volume, mm^3^	221.8 [134.6–312.5]	207.5 [134.5–293.2]	0.60
Plaque burden, %	38.6 ± 12.5	36.4 ± 11.0	0.080
*Composition of atherosclerosis*			
% FI volume	57.7 ± 10.5	60.4 ± 12.6	0.050
% FF volume	9.6 [6.0–13.7]	8.6 [5.8–12.2]	0.039
% NC volume	21.6 ± 8.0	20.8 ± 8.8	0.37
% DC volume	7.6 [4.7–13.9]	8.0 [4.3–13.3]	0.62
**(VH-)IVUS lesion parameters**			
≥1 Lesions, n (%)	120 (85.7)	123 (87.9)	0.73
Presence of high risk lesions, n (%)	90 (64.3)	83 (59.3)	0.46
High risk lesion type:			
*Degree of atherosclerosis*			
≥1 Lesion with plaque burden ≥70%, n (%)	31 (22.1)	27 (19.3)	0.65
≥1 Lesion with MLA ≤4.0mm^2^, n (%)	43 (30.7)	42 (30.0)	1.00
*Composition of atherosclerosis*			
≥1 TCFA, n (%)	57 (40.7)	57 (40.7)	1.00

FI, fibrous; FF, fibro-fatty; NC, necrotic core; DC, dense calcium; MLA, minimal lumen area; TCFA, thin-cap fibroatheroma.

Values are mean ± SD, median [interquartile range], or n (%).

P-values were obtained by paired samples t-test or McNemar test, whichever was appropriate.

The odds ratio (OR) (95% confidence interval (CI)) of current smoking for plaque burden above the median versus below the median was 1.69 (1.04–2.75), p = 0.033 (Table 4). After stratification on age, this tendency towards higher plaque burden in current smokers was only present in the lower age tertile (37.8±12.6% versus 33.9±11.1%, p = 0.09). However, the OR for plaque burden above the median versus below the median was not significant in this subgroup (S1 and S2 Tables). Furthermore, after stratification on indication for catheterization, plaque burden was significantly higher in current smokers presenting with ACS (38.3±12.8% versus 35.0±11.2%, p = 0.049 and OR 1.88 (1.02–3.44), p = 0.042) (S3 and S4 Tables).

**Table 4:** Odds ratios of current smoking for high plaque burden and for presence of high risk lesion types.

	OR (95% CI)	P-value
**(VH-)IVUS segment parameters**		
Plaque burden		
Below the median	1.00 (reference)	
Above the median	1.69 (1.04–2.75)	0.033
**(VH-)IVUS lesion parameters**		
≥1 Lesion with plaque burden ≥70%	1.20 (0.66–2.17)	0.55
≥1 Lesion with MLA ≤4.0mm^2^	1.03 (0.63–1.71)	0.90
≥1 TCFA	1.00 (0.63–1.60)	1.00

OR, odds ratio; CI, confidence interval; MLA, minimal lumen area; TCFA, thin-cap fibroatheroma.

P-values were obtained by conditional logistic regression.

The number of patients with ≥1 lesions was similar in current and never smokers (85.7% vs. 87.9%, p = 0.72) (Table 3). The odds ratio of having one or more lesions with plaque burden ≥70% was also similar (OR (95% CI): 1.47 (0.76–2.83)), as was the odds ratio of having one or more lesions with a minimal luminal area of ≤4.0 mm^2^ (Table 4). Subgroup analysis did not provide additional insights.

As described above, we found a borderline association with plaque burden, but no association with plaque volume. This seeming discrepancy may be due to the fact that plaque burden is not a direct measure of three dimensional plaque volume, but rather a two dimensional measure that also accounts for arterial wall remodeling. Specifically, the discrepancy may be explained by an association with negative remodeling. Therefore, we examined associations of smoking with remodeling in a post-hoc analysis. Smoking displayed a tendency toward a positive association with negative remodeling (OR (95% CI): 1.58 (0.89–2.81), p = 0.12), as well as a tendency toward a negative association with positive remodeling (OR (95% CI): 0.47 (0.21–1.05), p = 0.065).

### Composition of coronary atherosclerosis

**Fig 2:**
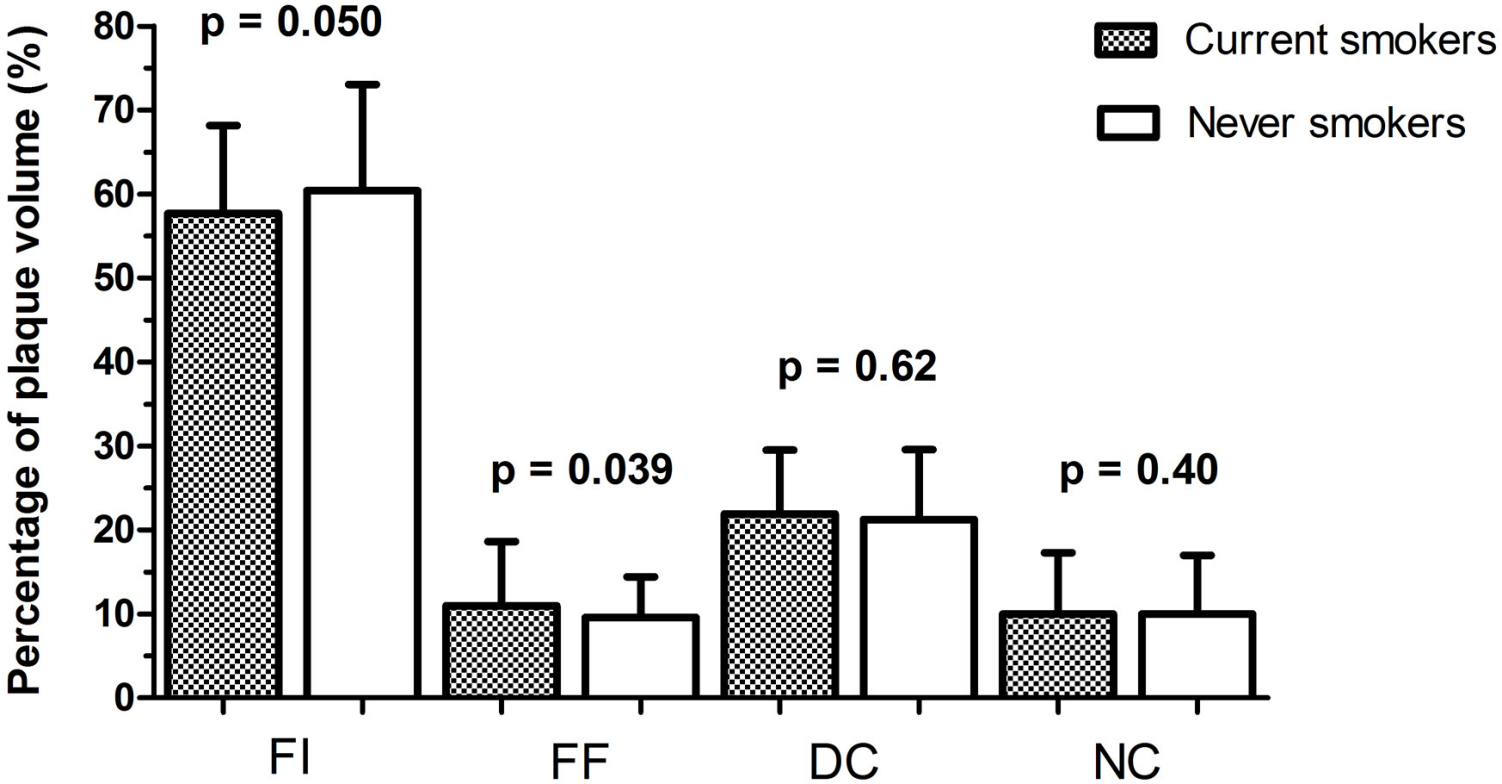
Difference in composition of coronary atherosclerosis between current and never smokers, after matching. FI = fibrous. FF = fibro-fatty. DC = dense calcium. NC = necrotic core.

VH-IVUS segment and lesion characteristics of the matched smokers and never smokers are listed in Table 3. Percentage of fibrous tissue (% FI) volume in the examined coronary segment tended to be lower in current smokers (57.7±10.5% vs. 60.4±12.6%, p = 0.050), which was driven by the lower age tertile (56.5±10.4% vs. 61.4±12.8%, p = 0.042) (S1 Table). Percentage of fibro-fatty tissue volume was higher in current smokers (9.6[6.0–13.7]% vs. 8.6[5.8–12.2]%, p = 0.039) (Table 3 and Fig 2), which was driven by the upper age tertile (11.1[6.3–15.3]% vs. 8.3[5.8–12.3], p = 0.08) (S1 Table). However, differences in percentage necrotic core (% NC) volume and dense calcium volume could not be demonstrated, and prevalence of ≥1 TCFA lesions was the same in current and never smokers (both 40.7%, Tables 3 and 4). After stratification on age, TCFA lesions tended to occur less often in current smokers in the upper age tertile (23.4% vs 44.7%, p = 0.06; OR 0.41 (0.17–0.99, p = 0.048) (S1 and S2 Tables).

## Discussion

This study investigated the associations of cigarette smoking with coronary atherosclerotic plaque burden, volume and composition as determined by (VH-)IVUS of a non-culprit section of a coronary artery in patients undergoing coronary angiography. Cigarette smoking showed a tendency towards higher coronary plaque burden, which was driven by an association in the subgroup of patients presenting with ACS. The magnitude of this effect was very modest. Furthermore, while smoking was associated with higher percentage of fibro-fatty plaque volume, no associations could be demonstrated with percentage necrotic core volume, nor with VH-IVUS derived TCFA lesions, suggesting that smoking has no major influence on plaque vulnerability.

Although several studies have examined the association between smoking and degree of coronary atherosclerosis as measured by IVUS [9–11,15–17], so far only one large study has applied virtual histology (VH-IVUS) to assess its association with composition of coronary atherosclerosis and plaque vulnerability. Philipp et al [10] found that smoking was not associated with plaque composition in a sample of 990 consecutive, non-selected patients, which is in line with our findings. However, they did not perform a lesion-based analysis, or a categorization into high-risk lesions such as TCFA, as we did. Missel et al [9] examined a subset of 473 male patients with de novo culprit coronary lesions from the same registry, and found a higher NC/DC ratio, a measure of plaque vulnerability, in smokers. Other VH parameters were not significantly influenced by smoking. A post-hoc analysis in our dataset showed no relation between smoking and NC/DC ratio (results not shown). In a small, underpowered study of 30 patients with stable angina Sano et al [11] found no association of plaque characteristics with smoking either. Remarkably, in our subgroup analysis, we found that TCFA lesions tended to occur less often in current smokers in the upper age tertile. A healthy survivor effect may pose a potential explanation for this finding.

With regard to degree of coronary atherosclerosis as assessed by IVUS, previous studies have rendered contradicting results. Nicholls et al [16] demonstrated that smoking was a weak independent predictor of percent plaque volume in 654 patients with a clinical indication for diagnostic coronary angiography. Furthermore, Von Birgelen et al [17] found an association between smoking and progression of plaque plus media cross-sectional area in 56 patients with de novo, hemodynamically nonsignificant plaques. In contrast, Kahlon et al concluded that smoking was not correlated with plaque burden in 897 consecutive patients undergoing IVUS investigation [15]. In our total study population, the association between smoking and plaque burden as well as plaque volume was not substantial. However, we did find such an association in the subgroup of patients presenting with ACS. In this subgroup, current smokers showed a higher plaque burden than never smokers. These findings concur with the fact that smoking is associated with both greater degrees of stenosis and an increased likelihood of acute plaque events [18], as well as with the fact that smoking is associated with reduced fibrinolytic potential and thus a pro-thrombotic phenotype [18]. Nevertheless, it should be recognized that the magnitude of the difference in degree of atherosclerosis between current and never smokers presenting with ACS was modest in our study, and that both pathophysiologic and clinical relevance of the findings may be questioned. Previous studies on smoking and degree of atherosclerosis on IVUS have not stratified their results on indication for angiography.

Our results do not support the hypothesis that smoking is associated with coronary plaque vulnerability. This may be explained by the possibility that plaque erosion, and not as much vulnerable plaque rupture, is the intermediate between smoking and cardiac adverse events. Histopathological studies have shown that luminal thrombosis may result from two different pathologies, namely plaque rupture and plaque erosion [19–22]. Plaque rupture seems to be highly associated with TCFAs and causes thrombotic coronary occlusion. Plaque erosion is characterized by an acute thrombus in direct contact with the intimal plaque, rich in smooth muscle cells with surrounding proteoglycan matrix and minimal inflammation. The lesions tend to be eccentric, infrequently calcified and cause less severe narrowing at sites of thrombosis [20,21]. Most eroded lesions have an absent or poorly defined necrotic core, which, when present, is not in close proximity to the luminal thrombus. Studies have shown that smoking is associated with plaque erosion and frequently causes coronary thrombosis [20,21]. This may possibly explain the general absence of an association of smoking with coronary plaque composition as assessed by VH-IVUS in the literature, and the inconsistent findings with regard to smoking and degree of coronary atherosclerosis as assessed by grayscale IVUS. We found that current smokers tend to have a slightly lower percentage fibrous plaque volume (driven by the lower age tertile), and that they have a somewhat higher percentage fibro-fatty plaque volume; however, this trend did not persist with regard to percentage necrotic core or presence of TCFA. These findings do not preclude plaque erosion as the underlying mechanism. Moreover, histopathological studies examining coronary arteries suggest that smoking predisposes patients to coronary thrombosis rather than promoting the progression of atherosclerosis [21,23,24]. These findings are supported by clinical patient studies showing that smokers seem to have a more favourable response to fibrinolytic therapy compared to nonsmokers, which may be attributed to their hypercoagulable state [25–27]. In the present study, we did not focus on the influence of smoking on blood coagulation.

Smoking displayed a tendency toward a positive association with negative remodeling, as well as a tendency toward a negative association with positive remodeling. A possible explanation for these seemingly counterintuitive findings lies in the interpretation of the early phases of remodeling. Modest positive lesion remodeling may be considered as a physiological, and thus favourable, response to progression of atherosclerotic plaque (also known as the Glagov adaptive phenomenon) [28]. In this light, smoking may point towards a lower adaptive capacity to atherosclerotic burden.

Some aspects of this study warrant consideration. A single non-culprit coronary vessel was imaged. This study design of ATHEROREMO-IVUS was based on the hypothesis that such a non-stenotic segment adequately reflects the state of the coronary wall of the larger coronary tree [12]. Both ex vivo and in vivo studies using IVUS in patients presenting with myocardial infarction have demonstrated the existence of additional TCFAs other than the culprit lesion in the culprit artery, as well as TCFAs in other arteries than the culprit artery [29]. Accordingly, the results of ATHEROREMO-IVUS, which we published earlier, have confirmed that the characteristics of the coronary wall of this non-culprit coronary vessel are strongly associated with subsequent cardiovascular outcome [30]. In addition, previous studies evaluating IVUS have similarly demonstrated that the coronary wall of comparable non-culprit, non-stenotic segments of a single vessel does reflect larger coronary disease burden and is associated with subsequent events [31,32]. Nevertheless, simultaneous assessment of the culprit vessel might have provided additional insights into the underlying disease mechanisms. Another limitation of this study is that IVUS is formally not capable of detecting the TCFA according to histopathological definitions [33,34]. Nonetheless, a concept of VH-IVUS derived TCFA has been postulated for plaques with a plaque burden ≥ 40% and a confluent necrotic core ≥ 10% in direct contact with the lumen in at least three VH-IVUS frames [14,33], and we have demonstrated earlier that such VH-IVUS derived TCFA lesions strongly and independently predict the occurrence of major adverse cardiac events within the current study population [30]. Furthermore, smoking status was determined by self-report in this cross-sectional study. To minimize the risk of misclassification, we excluded former smokers from our study. Finally, a matching procedure was necessary because of differences in baseline characteristics between smokers and never smokers. Since part of the smokers (n = 29) could not be matched to a never smoker, this study design entailed some loss of statistical power. Moreover, statistical power for the stratified analyses was limited.

In conclusion, we were not able to demonstrate a clear and strong association of cigarette smoking with degree of atherosclerosis and coronary plaque vulnerability as assessed by VH-IVUS in the current study. Additional studies, using various intravascular imaging modalities, are needed to further describe the association between smoking and in-vivo degree and composition of coronary plaque, and to herewith discern the mechanisms underlying the association between smoking and cardiac adverse events.

## Supporting Information

S1 File Database supporting information file. (SAV)

S1 Table (VH-)IVUS segment and lesion characteristics in the matched set, stratified on age. (DOCX)

S2 Table Odds ratios of current smoking for high plaque burden and for presence of high risk lesion types, stratified on age. (DOCX)

S3 Table (VH-)IVUS segment and lesion characteristics in the matched set, stratified on indication. (DOCX)

S4 Table Odds ratios of current smoking for high plaque burden and for presence of high risk lesion types, stratified on indication. (DOCX)
