# Supplementary material for: Smoking in Relation to Coronary Atherosclerotic Plaque Burden, Volume and Composition on Intravascular Ultrasound
Source: PLoS One. 2015 Oct 22;10(10):e0141093. doi: 10.1371/journal.pone.0141093 (PMC4619630; doi:10.1371/journal.pone.0141093)
Supplement: S1 Table — (DOCX) [file pone.0141093.s002.docx]

**S1 Table. (VH-)IVUS segment and lesion characteristics in the matched set, stratified on age**

|  | **Lower age tertile [34.12 – 52.96 years]** | | |  | **Middle age tertile [53.07 – 61.68 years]** | | |  | **Upper age tertile [61.73 – 85.03 years]** | | |
| --- | --- | --- | --- | --- | --- | --- | --- | --- | --- | --- | --- |
|  | **Current**  **smokers** | **Never**  **smokers** | ***P*** |  | **Current**  **smokers** | **Never**  **smokers** | ***P*** |  | **Current**  **smokers** | **Never**  **smokers** | ***P*** |
|  | (n = 46) | (n = 46) |  |  | (n = 47) | (n = 47) |  |  | (n = 47) | (n = 47) |  |
| Age, mean±SD | 47.5 ± 4.6 | 47.9 ± 4.8 | 0.12 |  | 57.4 ± 2.7 | 57.5 ± 2.8 | 0.27 |  | 68.6 ± 5.4 | 68.6 ± 5.3 | 0.93 |
| **(VH-)IVUS segment parameters** |  |  |  |  |  |  |  |  |  |  |  |
| Segment length, mm | 45.7 ± 13.1 | 44.3 ± 12.8 | 0.64 |  | 44.6 ± 17.8 | 45.2 ± 12.5 | 0.85 |  | 46.0 ± 15.2 | 44.5 ± 14.5 | 0.63 |
| *Degree of atherosclerosis* |  |  |  |  |  |  |  |  |  |  |  |
| Plaque volume, mm^3^ | 235.5 [127.8 – 322.3] | 192.0 [116.9 – 266.8] | 0.51 |  | 200.9 [140.8 – 309.9] | 214.3 [134.1 – 312.5] | 0.59 |  | 226.0 [147.7 – 306.8] | 217.0 [142.8 – 334.2] | 0.74 |
| Plaque burden, % | 37.8 ± 12.6 | 33.9 ± 11.1 | 0.09 |  | 38.6 ± 12.7 | 37.7 ± 10.8 | 0.67 |  | 39.5 ± 12.6 | 37.4 ± 10.9 | 0.38 |
| *Composition of atherosclerosis* |  |  |  |  |  |  |  |  |  |  |  |
| % FI volume | 56.5 ± 10.4 | 61.4 ± 12.8 | 0.042 |  | 58.6 ± 10.0 | 59.0 ± 12.4 | 0.89 |  | 57.8 ± 11.1 | 60.8 ± 12.8 | 0.22 |
| % FF volume | 8.4 [4.8 – 11.6] | 9.4 [6.0 – 12.1] | 0.69 |  | 9.7 [6.1 – 12.7] | 8.8 [5.5 – 12.8] | 0.14 |  | 11.1 [6.3 – 15.3] | 8.3 [5.8 – 12.3] | 0.08 |
| % NC volume | 23.2 ± 9.3 | 21.0 ± 9.6 | 0.23 |  | 21.6 ± 7.0 | 21.1 ± 8.8 | 0.77 |  | 20.1 ± 7.4 | 20.3 ± 8.0 | 0.98 |
| % DC volume | 7.4 [4.9 – 13.1] | 7.7 [3.3 – 10.4] | 0.28 |  | 6.8 [4.5 – 14.1] | 9.1 [4.4 – 14.5] | 0.59 |  | 8.9 [4.5 – 14.8] | 8.0 [5.0 – 15.0] | 0.68 |
|  |  |  |  |  |  |  |  |  |  |  |  |
| **(VH-)IVUS lesion parameters** |  |  |  |  |  |  |  |  |  |  |  |
| ≥1 Lesions, n (%) | 39 (84.8) | 37 (80.4) | 0.79 |  | 41 (87.2) | 43 (91.5) | 0.75 |  | 40 (85.1) | 43 (91.5) | 0.51 |
| Presence of high risk lesions, n (%) | 33 (71.7) | 25 (54.3) | 0.15 |  | 28 (59.6) | 27 (57.4) | 1.00 |  | 29 (61.7) | 31 (66.0) | 0.83 |
| High risk lesion type: |  |  |  |  |  |  |  |  |  |  |  |
| *Degree of atherosclerosis* |  |  |  |  |  |  |  |  |  |  |  |
| ≥1 Lesion with plaque burden ≥70%, n (%) | 10 (21.7) | 6 (13.0) | 0.34 |  | 7 (14.9) | 11 (23.4) | 0.39 |  | 14 (29.8) | 10 (21.3) | 0.52 |
| ≥1 Lesion with MLA ≤4.0mm^2^, n (%) | 15 (32.6) | 12 (26.1) | 0.63 |  | 11 (23.4) | 16 (34.0) | 0.38 |  | 17 (36.2) | 14 (29.8) | 0.68 |
| *Composition of atherosclerosis* |  |  |  |  |  |  |  |  |  |  |  |
| ≥1 TCFA, n (%) | 26 (56.5) | 20 (43.5) | 0.29 |  | 20 (42.6) | 16 (34.0) | 0.54 |  | 11 (23.4) | 21 (44.7) | 0.06 |
