# Supplementary material for: Smoking in Relation to Coronary Atherosclerotic Plaque Burden, Volume and Composition on Intravascular Ultrasound
Source: PLoS One. 2015 Oct 22;10(10):e0141093. doi: 10.1371/journal.pone.0141093 (PMC4619630; doi:10.1371/journal.pone.0141093)
Supplement: S2 Table — (DOCX) [file pone.0141093.s003.docx]

**S2 Table. Odds ratios of current smoking for high plaque burden and for presence of high risk lesion types, stratified on age**

|  | **Lower age tertile [34.12 – 52.96 years]** | |  | **Middle age tertile [53.07 – 61.68 years]** | |  | **Upper age tertile [61.73 – 85.03 years]** | |
| --- | --- | --- | --- | --- | --- | --- | --- | --- |
|  | **OR (95% CI)** | ***P*** |  | **OR (95% CI)** | ***P*** |  | **OR (95% CI)** | ***P*** |
|  |  |  |  |  |  |  |  |  |
| **(VH-)IVUS segment parameters** |  |  |  |  |  |  |  |  |
| Plaque burden |  |  |  |  |  |  |  |  |
| Below the median | 1.00 (reference) |  |  | 1.00 (reference) |  |  | 1.00 (reference) |  |
| Above the median | 1.67 (0.73 – 3.81) | 0.23 |  | 1.44 (0.62 – 3.38) | 0.40 |  | 2.00 (0.86 – 4.67) | 0.11 |
|  |  |  |  |  |  |  |  |  |
| **(VH-)IVUS lesion parameters** |  |  |  |  |  |  |  |  |
| ≥1 Lesion with plaque burden ≥70% | 2.33 (0.60 – 9.02) | 0.22 |  | 0.50 (0.15 – 1.66) | 0.26 |  | 1.44 (0.62 – 3.38) | 0.40 |
| ≥1 Lesion with MLA ≤4.0mm^2^ | 1.43 (0.54 – 3.75) | 0.47 |  | 0.62 (0.26 – 1.49) | 0.28 |  | 1.30 (0.57 – 2.97) | 0.53 |
| ≥1 TCFA | 1.75 (0.73 – 4.17) | 0.21 |  | 1.40 (0.62 – 3.15) | 0.42 |  | 0.41 (0.17 – 0.99) | 0.048 |
