# Supplementary material for: Smoking in Relation to Coronary Atherosclerotic Plaque Burden, Volume and Composition on Intravascular Ultrasound
Source: PLoS One. 2015 Oct 22;10(10):e0141093. doi: 10.1371/journal.pone.0141093 (PMC4619630; doi:10.1371/journal.pone.0141093)
Supplement: S3 Table — (DOCX) [file pone.0141093.s004.docx]

|  | **ACS patients** | | |  | **SAP patients** | | |
| --- | --- | --- | --- | --- | --- | --- | --- |
|  | **Current**  **smokers** | **Never**  **smokers** | ***P*** |  | **Current**  **smokers** | **Never**  **smokers** | ***P*** |
|  | (n = 96) | (n = 96) |  |  | (n = 44) | (n = 44) |  |
|  |  |  |  |  |  |  |  |
| **(VH-)IVUS segment parameters** |  |  |  |  |  |  |  |
| Segment length, mm | 44.9 ± 14.7 | 43.8 ± 14.0 | 0.63 |  | 46.7 ± 17.0 | 46.5 ± 11.1 | 0.97 |
| *Degree of atherosclerosis* |  |  |  |  |  |  |  |
| Plaque volume, mm^3^ | 215.7 [131.0 – 312.5] | 199.7 [113.6 – 282.2] | 0.36 |  | 226.8 [146.6 – 315.3] | 224.9 [148.4 – 327.5] | 0.61 |
| Plaque burden, % | 38.3 ± 12.8 | 35.0 ± 11.2 | 0.049 |  | 39.3 ± 12.1 | 39.3 ± 9.9 | 0.97 |
| *Composition of atherosclerosis* |  |  |  |  |  |  |  |
| % FI volume | 58.1 ± 9.8 | 61.1 ± 13.1 | 0.08 |  | 56.8 ± 11.9 | 58.9 ± 11.4 | 0.39 |
| % FF volume | 9.2 [5.5 – 13.2] | 8.6 [5.8 – 12.0] | 0.14 |  | 10.6 [7.5 – 14.4] | 9.3 [5.7 – 12.7] | 0.14 |
| % NC volume | 22.0 ± 8.1 | 20.8 ± 9.3 | 0.34 |  | 20.1 ± 7.8 | 20.7 ± 7.5 | 0.91 |
| % DC volume | 7.8 [4.9 – 13.4 ] | 7.5 [4.3 – 11.8] | 0.42 |  | 7.6 [3.9 – 16.0] | 9.4 [4.5 – 16.5] | 0.76 |
|  |  |  |  |  |  |  |  |
| **(VH-)IVUS lesion parameters** |  |  |  |  |  |  |  |
| ≥1 Lesions, n (%) | 84 (87.5) | 82 (85.4) | 0.84 |  | 36 (81.8) | 41 (93.2) | 0.18 |
| Presence of high risk lesions, n (%) | 61 (63.5) | 60 (62.5) | 1.00 |  | 29 (65.9) | 23 (52.3) | 0.26 |
| High risk lesion type: |  |  |  |  |  |  |  |
| *Degree of atherosclerosis* |  |  |  |  |  |  |  |
| ≥1 Lesion with plaque burden ≥70%, n (%) | 21 (21.9) | 15 (15.6) | 0.38 |  | 10 (22.7) | 12 (27.3) | 0.77 |
| ≥1 Lesion with MLA ≤4.0mm^2^, n (%) | 26 (27.1) | 29 (30.2) | 0.75 |  | 17 (38.6) | 13 (29.5) | 0.52 |
| *Composition of atherosclerosis* |  |  |  |  |  |  |  |
| ≥1 TCFA, n (%) | 38 (39.6) | 42 (43.8) | 0.66 |  | 19 (43.2) | 15 (34.1) | 0.54 |

**S3 Table. (VH-)IVUS segment and lesion characteristics in the matched set, stratified on indication**
