# Supplementary material for: Smoking in Relation to Coronary Atherosclerotic Plaque Burden, Volume and Composition on Intravascular Ultrasound
Source: PLoS One. 2015 Oct 22;10(10):e0141093. doi: 10.1371/journal.pone.0141093 (PMC4619630; doi:10.1371/journal.pone.0141093)
Supplement: S4 Table — (DOCX) [file pone.0141093.s005.docx]

**S4 Table. Odds ratios of current smoking for high plaque burden and for presence of high risk lesion types, stratified on indication**

|  | **ACS patients** | |  | **SAP patients** | |
| --- | --- | --- | --- | --- | --- |
|  | **OR (95% CI)** | ***P*** |  | **OR (95% CI)** | ***P*** |
|  |  |  |  |  |  |
| **(VH-)IVUS segment parameters** |  |  |  |  |  |
| Plaque burden |  |  |  |  |  |
| Below the median | 1.00 (reference) |  |  | 1.00 (reference) |  |
| Above the median | 1.88 (1.02 – 3.44) | 0.042 |  | 1.40 (0.62 – 3.15) | 0.42 |
|  |  |  |  |  |  |
| **(VH-)IVUS lesion parameters** |  |  |  |  |  |
| ≥1 Lesion with plaque burden ≥70% | 1.46 (0.72 – 2.96) | 0.29 |  | 0.71 (0.23 – 2.25) | 0.57 |
| ≥1 Lesion with MLA ≤4.0mm^2^ | 0.86 (0.46 – 1.61) | 0.63 |  | 1.44 (0.62 – 3.38) | 0.40 |
| ≥1 TCFA | 0.84 (0.47 – 1.50) | 0.56 |  | 1.40 (0.62 – 3.15) | 0.42 |
